# Physical functioning trajectories over statutory retirement: a finnish occupational cohort study

**DOI:** 10.1186/s13690-024-01483-2

**Published:** 2025-01-10

**Authors:** Pauliina Saha, Jatta Salmela, Aapo Hiilamo, Anna Liisa Aho, Tea Lallukka

**Affiliations:** 1 Health Sciences Unit, Tampere University, Tampere, Finland; 2 Department of Public Health, University of Helsinki, Helsinki, Finland; 3 Max Planck Institute for Demographic Research, Rostock, Germany

**Keywords:** Physical functioning, Workload, Retirement, Trajectory analysis, Ageing

## Abstract

**Background:**

The association of workload and performance with physical functioning is recognised among the ageing public sector workforce. The characteristics of working conditions and social- and health-related factors associated with physical functioning after statutory retirement are still unknown. Also, previous studies on changes in physical functioning have not used a person-oriented approach. We examined physical functioning trajectories over statutory retirement and how social- and health-related factors are associated with them. Our aim was to identify distinct developmental trajectories of physical functioning over statutory retirement and to examine how social- (age, gender, marital status, education) and health-related (physical workload, self-reported sleep problems, alcohol consumption, smoking, fruit and vegetable (F&V) consumption, leisure-time physical activity (LTPA), and body mass index (BMI)) factors before retirement were associated with the identified trajectories.

**Methods:**

We used data from the Helsinki Health Study cohort. Participants consisted of 2736 employees of the City of Helsinki, Finland who retired during the follow-up. Growth mixture modelling was used to identify physical functioning trajectories and multinominal regression analyses to examine associations of social- and health-related factors with them.

**Results:**

Three distinct developmental patterns in physical functioning before and after retirement were found among ageing and retired employees. Lower educational level, sleep problems, physical inactivity, and obesity were associated with the trajectory groups of ‘fast decreasing’ and ‘slowly increasing’, compared to the ‘stable high’ trajectory.

**Conclusion:**

The results suggest that poor social- and health-related factors are key risk factors associated with declining and lower-level physical functioning over the retirement period. Supporting healthy lifestyles among older employees might maintaining good physical functioning until retirement and beyond.

**Supplementary Information:**

The online version contains supplementary material available at 10.1186/s13690-024-01483-2.

**Table:** 

**Text box 1. Contributions to the literature**	
• The novel contributions of this research are the examined trajectories in physical functioning after statutory retirement, and the covariates behind these identified trajectories.	
• This study shows that lower education and health-related risk factors were associated with trajectories of lower levels of physical functioning before retirement and declining physical functioning after statutory retirement.	
• Future studies could investigate whether changes in these or other social- and health-related factors during the retirement period impact the trajectories of physical functioning after retirement.	

## Background

Population ageing started in Europe several decades ago and is still a long-term trend which reduces the proportion of the working-age population [1]. The share of older workers in the employment force has already substantially grown, but there is still the potential for this rate to increase even further [2]. To lengthen working careers, work should be sustainably arranged, that is, health-impairing conditions should be reduced and health-promoting ones supported.

Current empirical evidence on changes in physical functioning after retirement is ambiguous [3]. Some studies suggest that physical functioning declines shortly after retirement [4–8], while other studies claim that it improves [9–13]. By far, there is a lack of evidence on how physical functioning develops after statutory retirement and whether workload and social- and health-related factors are associated with physical functioning trajectories over statutory retirement. It is known that physical functioning deteriorates along with ageing [14, 15], but it remains unclear if there are distinct developmental patterns in physical functioning around retirement. While the statutory retirement age varies little among municipal employees, it is challenging to distinguish between changes that occur due to ageing and retirement. It is suggested that future studies could focus on the issues behind the changes in functioning after retirement, for example, the mechanisms and associations between health-related factors and changing functioning after retirement [3]. This would provide new information and help evaluate the abilities to operate around retirement, and then possibly work even longer than nowadays. 

The association of workload and performance with physical functioning is recognised among the ageing public sector workforce [16, 17]. Difficulties in functioning are relatively uncommon among statutory retirees, but after the age of 75, the number of difficulties in several daily activities seems to grow significantly. Demographic differences are also recognised, such as socioeconomic and gender-related differences [18]. It is also recognised that difficulties in physical functioning after retirement are associated with social- and health-related risks [5]. The characteristics of working conditions and social- and health-related factors associated with physical functioning after statutory retirement are still unknown. Also, previous studies on changes in physical functioning have not used person-oriented approaches, which can identify latent groups of people who have rather similar pathways in how their physical functioning develops over time.

## Methods

The aim of this study is to identify developmental patterns in physical functioning before and after retirement and the key covariates associated with them. To the best of our knowledge, this is the first study examining the trajectory groups of physical functioning among statutory retirees with a person-oriented approach. Our additional aim is to identify possible determinants of belonging to a certain trajectory group. The specific research questions are:

1. What kind of developmental trajectories of physical functioning can be identified over statutory retirement?
2. How are social- and health-related factors before retirement associated with the identified physical functioning trajectories?

### Data

We used data from the Helsinki Health Study cohort, which has examined health and its determinants of around 9000 employees of the City of Helsinki, Finland, since 2000 [19]. The baseline survey was conducted in 2000–2002 among employees aged 40, 45, 50, 55, and 60 years (n = 8960, response rate 67%). Follow-up surveys were conducted in 2007 (*n* = 7332, response rate 83%), 2012 (*n* = 6814, response rate 79%), and 2017 (*n* = 6832, response rate 82%). At baseline, all responders were working, and by Phase 4, 70% of the responders had retired. The follow-up surveys were mailed to all responders of the phase 1 study regardless of where they currently worked or whether they had already retired. The target population of this study was the employees who statutorily retired during the follow-up. We included only participants with information on physical functioning from at least three survey phases. The follow-up surveys included a question about the timing of retirement (year and month), and a question about the type of retirement. The participants who had missing information on the timing of retirement (*n* = 449) or who had unclear information on the timing of retirement (n = 7) were excluded. The final analytical sample consisted of 2736 responders (80% women, corresponding well to the target population [19]). The participant selection criteria are described in the Supplementary Fig. 1.

The data cannot be made publicly available due to strict data protection laws, but access to data can be applied from the Helsinki Health Study group upon reasonable request and following the data sharing policy and data protection laws and regulations. The study protocol has been approved by the ethics committees of the Department of Public Health, University of Helsinki, and the health authorities of the City of Helsinki.

## Measures

### Physical functioning

Physical functioning was measured using the RAND-36 questionnaire. The RAND-36 questionnaire is one of the most widely used survey instruments to measure health-related quality of life, consisting of 36 items with 8 dimensions of health. The dimensions are physical functioning, role limitations caused by physical health problems, role limitations caused by emotional problems, social functioning, emotional well-being, energy/fatigue, pain, and general health perceptions. We used dimension of physical functioning [20, 22]. The physical functioning subscale consists of ten items, for instance, vigorous activities (e.g., running), moderate activities (e.g., brisk walking), lifting and carrying groceries, climbing stairs, and how the health of the respondent limits these activities. Each item had three response alternatives: ‘yes, limited a lot’, ‘yes, limited a little’ and ‘no, not limited at all’. Further details are found elsewhere [20]. Physical functioning scores were derived from the dimension of physical health, range between 0–100. Higher scores indicate better functioning [21, 22].

### Social- and health-related factors

Age (continuous), gender (man/woman), marital status (married/cohabiting and other), and responder’s own education, were derived from the phase 1 questionnaire. Education was classified into three groups: higher education (university degree or more), intermediate education (matriculation or college education), and basic education (primary or secondary school or less).

Physical workload, self-reported sleep problems, alcohol consumption, smoking, fruit and vegetable (F&V) consumption, leisure-time physical activity (LTPA), and body mass index (BMI) were derived from the phase prior to a respondent's retirement. Physical working conditions were inquired by a single-item question about the physical strenuousness of work. The question included four response alternatives: ‘very light’, ‘rather light’, ‘rather strenuous’ and ‘very strenuous’. To follow previous studies [23, 24], this was further classified into three groups: physically non-strenuous (very light), intermediate (rather light), and strenuous (rather strenuous/very strenuous) work. Sleep problems were measured using a 4-item version of the Jenkins questionnaire [23]. Each item includes 6 response choices varying from no sleep problems at all to having sleep problems in 22–28 nights/month.) The questionnaire evaluates the frequency of certain sleep problems: difficulty falling asleep, frequent awakenings during the night, trouble remaining asleep, and subjective feelings of fatigue and sleepiness despite receiving a typical night’s rest [25, 26]. Participants were divided into three groups: having no, occasional (any symptoms in ≤ 14 nights/month), and frequent (any symptoms in > 14 nights/month) sleep problems. For LTPA, weekly metabolic equivalent task (MET) hours were formed based on the self-reported information on the participants’ average weekly hours of LTPA within the past 12 months, including commuting to work. These were collected in four grades of intensity: walking, brisk walking, jogging and running, or their equivalent activities. LTPA was divided into three groups: vigorous activity (≥ 14 MET hours/week including the two highest intensity grades), moderate activity (≥ 14 MET hours/week including the two lowest intensity grades), and inactivity (< 14 MET hours/week) [27, 28]. Alcohol consumption was assessed by asking how often the responder drinks six or more units of alcohol on a single occasion. Frequency alternatives varied from ‘never’ to ‘daily or almost daily’. Alcohol consumption was dichotomised into having no binge drinking (once a month or less) and having binge drinking (once a week or more) behaviour. Smoking status was dichotomised into ‘no’ and ‘yes’, assessing this by asking if responders currently smoked cigarettes, cigars or pipe tobacco (‘yes’/ ‘no’). F&V consumption was derived from a 20-item food frequency questionnaire (FFQ) which assessed the frequency of consumed food items during the past four weeks. This included seven frequency alternatives from ‘not at all’ to ‘twice a day or more’. F&V consumption was dichotomised into daily (consuming both F and V daily) and non-daily consumption (F or V less than daily). BMI was computed using self-reported weight and height (kg/m^2^). BMI was divided into three groups: normal weight (BMI 18.5–24.9 kg/m^2^), overweight (BMI 25.0–29.9kg/m^2^), and obesity (BMI ≥ 30.0 kg/m^2^).

Since there was only a small number of missing values (0–6%) in most covariates, missing data were merged in these cases with ‘the most favourable’ group of a covariate, which is a conservative way of handling missing data. We completed a complete case analysis, and we also analysed missing data as its own group (data not shown). Merging missing values to the most favourable group was the most suitable option since the other analyses caused widening confidence intervals (and thus, increased uncertainty in the estimates). Additionally, complete case analyses did not essentially change the results. Consequently, we merged missing values in education (0.8%) to higher education, in marital status (0.3%) to married/cohabiting, in BMI (1.1%) to normal weight, in F&V consumption (1.4%) to daily, in alcohol consumption (2.4%) to no binge-drinking, in smoking (1.1%) to no, and in physical workload (5.7%) to non-strenuous. The proportion of missing values in sleep problems and LTPA was higher (8.8% and 9.6%, respectively), thus we kept missing values in their own groups in these covariates. Sensitivity analyses also showed that merging missing values with the most favourable groups in these two covariates biased the results and yielded widening CIs (data not shown).

### Statistical analyses

Statistical methods included cross-tabulations with χ2 tests, growth mixture modelling (GMM), and multinomial logistic regression analysis. We identified physical functioning trajectories using GMM, which identifies multiple unobserved sub-populations and examines differences in change within these populations [29, 30]. We used the R package LCMM to identify the trajectories [31]. The x-axis presents the timing of retirement, showing how physical functioning develops before and after retirement, with the zero point indicating the retirement year. The follow-up period varied from 10 to 17 years, and the mean follow-up period was 11 years. We performed GMM analyses with two to four latent class solutions (Supplementary Table 1). Bayesian Information Criterion (BIC), Akaike Information Criterion (AIC), the average of posterior probabilities of group membership (> 0.7), and the size of each trajectory group (> 5% of participants or n > 100) were used as statistical criteria to select the most fitted model [32]. In addition, the interpretability of the trajectories was considered in the model selection. Each participant was assigned to the trajectory group for which they had the highest group membership probability. The statistics of model fit are presented in Supplementary Table 1. The statistical power was not sufficient for gender-stratified analyses.

We cross-tabulated the selected trajectory groups with the covariates and then performed the regression models. We first adjusted the regression model for age and gender (model 1), and then further for marital status, education, and physical workload (model 2). Finally, we added health-related factors (i.e., sleep problems, LTPA, alcohol consumption, smoking, F&V consumption, and BMI) to model 2 (model 3). We show the results from the regression analyses as odds ratios (OR) with their 95% confidence intervals (CI), and the complete results are included in the supplementary materials (Supplementary Table 2). All statistical analyses, except trajectory modelling, were conducted using IBM SPSS version 25.

## Results

Descriptive statistics of the participants are shown in Supplementary Table 3. The mean age of participants at the phase prior to retirement was 60.1 years. The average physical functioning scores were higher among men than women through the follow-up. Most participants had no binge drinking behaviour (91%) and were not smokers (88%), whereas only a small number of participants did not have any sleep problems (11%).

**Fig. 1:**
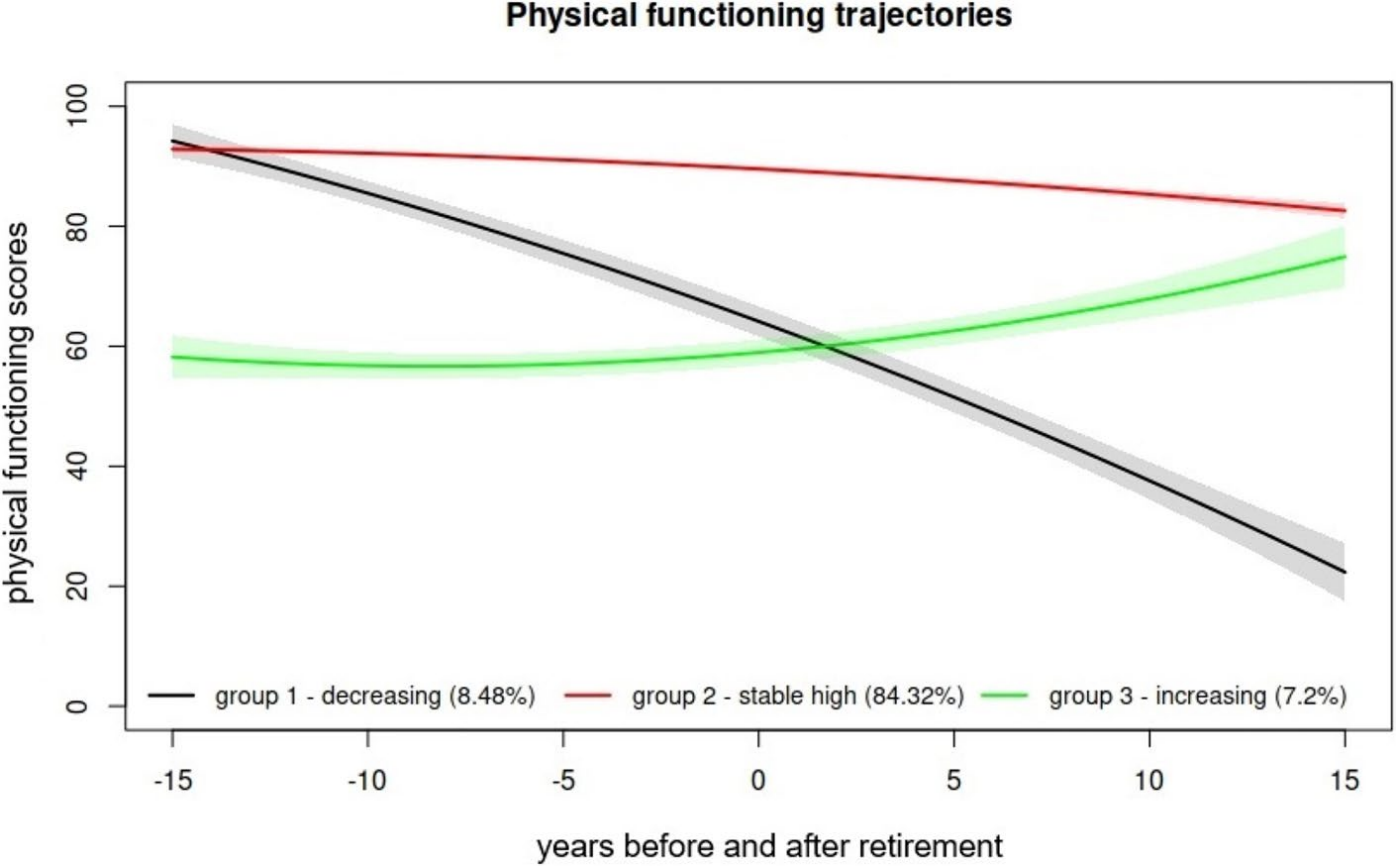
Physical functioning trajectories with 95% confidence intervals and their prevalence (%), identified by growth mixture modelling

We selected a trajectory model with three physical functioning trajectories (Fig. 1). The first trajectory group ‘fast decreasing’ included 8.5% (*n* = 232) of the study population, the second group ‘stable high’ 84.3% (*n* = 2307), and the third group ‘slowly increasing’ 7.2% (*n* = 197). The ‘fast decreasing’ trajectory started with high physical functioning scores but turned steeply to relatively low physical functioning scores during the follow-up. The ‘stable high’ trajectory remained relatively high during the whole follow-up. The ‘slowly increasing’ trajectory started from lower physical functioning scores than the other two trajectory groups but increased over the follow-up, slightly more steeply after than prior to retirement.

Group means and fitted lines with 95% confidence intervals are shown. Group 1 ‘fast decreasing’, group 2 ‘stable high’, group 3 ‘slowly increasing’. X-axis shows years before and after retirement, 0 indicating the retirement year.

The proportion of male employees was highest in the trajectory group ‘stable high’ compared to the other groups, while the proportional share of female employees was highest in the trajectory group ‘slowly increasing’ (Table 1). Higher education was more common in the trajectory group ‘stable high’ (34%) than in the groups ‘slowly increasing’ (16%) and ‘fast decreasing’ (22%). Physically strenuous workloads were less common in the trajectory group ‘stable high’ (29%) compared to the other two groups (‘slowly increasing’ 41%; ‘fast decreasing’ 39%). A smaller proportion reported no sleep problems in the trajectory group ‘slowly increasing’ (2%) compared to the groups ‘fast decreasing’ (5%) and ‘stable high’ (12%). Vigorous LTPA was more common in the trajectory group ‘stable high’ (22%) than in the groups ‘slowly increasing’ (7.1%) and ‘fast decreasing’ (8.2%). Obesity was less typical in the trajectory group ‘stable high’ (12%) than in the other two groups (‘fast decreasing’ 38%; ‘slowly increasing’ 36%).

**Table 1:** Background characteristics of the study population by physical functioning trajectory groups (n, %)

	Group 1—fast decreasing	Trajectory group	Group 3—slowly increasing	Chi-Squared test, *p*-value
		Group 2—stable high		
**Age (mean, standard deviation)**	60.32 2.325	60.07 2.581	60.31 2.838	- -
**Gender**				< 0.001
Women	199 (85.8)	1809 (78.4)	178 (90.4)	
Men	33 (14.2)	498 (21.6)	19 (9.6)	
**Marital status**				0.012
Married/cohabiting	144 (62.1)	1613 (69.9)	125 (63.5)	
Other	88 (37.9)	694 (30.1)	72 (36.5)	
**Education**^**a**^				< 0.001
Higher education	51 (22.0)	776 (33.6)	32 (16.2)	
Intermediate education	116 (50.0)	1079 (46.8)	108 (54.8)	
Basic education	65 (28.0)	452 (19.6)	57 (28.9)	
**Physical workload**^**b**^				< 0.001
Physically non-strenuous	60 (25.9)	667 (28.9)	50 (25.4)	
Intermediate (rather light)	82 (35.3)	966 (41.9)	67 (34.0)	
Physically strenuous (rather strenuous/very strenuous)	90 (38.8)	674 (29.2)	80 (40.6)	
**Sleep problems**^**c**^				< 0.001
No	12 (5.2)	273 (11.8)	3 (1.5)	
Occasional	156 (67.2)	1654 (71.7)	146 (74.1)	
Frequent	40 (17.2)	179 (7.8)	31 (15.7)	
Missing	24 (10.3)	201 (8.7)	17 (8.6)	
**Leisure-time physical activity (LTPA)**^**d**^				< 0.001
Vigorously active	19 (8.2)	515 (22.3)	14 (7.1)	
Moderately active	53 (22.8)	662 (28.7)	48 (24.4)	
Inactive	138 (59.5)	900 (39.0)	124 (62.9)	
Missing	22 (9.5)	230 (10.0)	11 (5.6)	
**Alcohol consumption**^**e**^				0.003
No binge-drinking	197 (84.9)	2113 (91.6)	177 (89.8)	
Binge-drinking	35 (15.1)	194 (8.4)	20 (10.2)	
**Smoking**^**f**^				0.001
No	186 (80.2)	2042 (88.5)	173 (87.8)	
Yes	46 (19.8)	265 (11.5)	24 (12.2)	
**Fruit and vegetable consumption**^**g**^				0.001
Daily	119 (51.3)	1460 (63.3)	119 (60.4)	
Non-daily	113 (48.7)	847 (36.7)	78 (39.6)	
**Body mass index (BMI)**				< 0.001
Normal weight (BMI < 25 kg/m^2^)	66 (28.4)	1123 (48.7)	48 (24.4)	
Overweight (BMI 25.0–30 kg/m^2^)	77 (33.2)	900 (39.0)	78 (39.6)	
Obesity (BMI ≥ 30 kg/m^2^)	89 (38.4)	284 (12.3)	71 (36.0)	

^a^ Higher education = university degree or more, intermediate education = matriculation or college education, basic education = primary or secondary school or less

^b^ Physically non-strenuous = very light, Intermediate = rather light, physically strenuous = rather strenuous/very strenuous

^c^ Occasional = any symptoms in ≤ 14 nights/month; frequent = any symptoms in > 14 nights/month

^d^ Vigorous activity = ≥ 14 metabolic equivalent task (MET) hours/week including the two highest intensity grades, moderate activity = ≥ 14 MET hours/week including the two lowest intensity grades, inactivity = < 14 MET hours/week

^e^ Binge-drinking = once a week or more, no binge-drinking = once a month or less

^f^ No = not currently smoking, yes = currently smoking

^g^ Daily consumption = consuming both fruit and vegetables daily, non-daily consumption = consuming fruit or vegetables less than daily

The associations of the covariates with physical functioning trajectories are shown in Table 2. The ‘stable high’ trajectory group was used as a reference. Men had smaller odds of belonging to the ‘fast decreasing’ (0.60, 95% CI 0.41–0.89) or ‘slowly increasing’ (0.39, 95% CI 0.24–0.63) groups (model 1), which remained with further adjustments (model 2 and 3). Participants with basic education had an increased odds of belonging to the ‘fast decreasing’ (OR 2.10, 95% CI 1.43–3.09) and ‘slowly increasing’ trajectory groups (OR 2.89 95% CI 1.83–4.50) when adjusting the analyses for age and gender (model 1), and the associations remained after further adjustments (models 2 and 3). Those reporting occasional or frequent sleep problems had higher odds of belonging to the trajectory groups ‘fast decreasing’ (OR 2.11, 95% CI 1.15–3.84; OR 4.90, 95% CI 2.50–9.62, correspondingly) and ‘slowly increasing’ (OR 7.75, 95% CI 2.45–24.49; OR 14.76, 95% CI 4.44–49.06, correspondingly) (model 1). The associations slightly decreased but remained strong after further adjustments (models 2 and 3). Those reporting physical inactivity had higher odds of belonging to the trajectory groups ‘fast decreasing’ (OR 3.97 95% CI 2.42–6.501.86–3.85) and ‘slowly increasing’ (OR 2.45 95% CI 1.33–4.49) (model 1). The associations remained after adjusting model 1 further for marital status, education, and physical workload (model 2), but decreased after adjusting the models further for health-related factors (model 3). Participants with obesity had higher odds of belonging to the trajectory groups ‘fast decreasing’ (OR 5.44 95% CI 3.85–7.68) and ‘slowly increasing’ (OR 6.06 95% CI 4.10–8.97), and the associations only slightly decreased after further adjustments (models 2 and 3).

**Table 2:** Associations of social- and health-related factors with physical functioning trajectories (ref. ‘stable high’ trajectory group)^a^

	**Model 1**^**b**^		**Model 2**^**c**^		**Model 3**^**d**^	
	**Group 1—fast decreasing**	**Group 3—slowly increasing**	**Group 1—fast decreasing**	**Group 3—slowly increasing**	**Group 1—fast decreasing**	**Group 3—slowly increasing**
	**OR (95% CI)**	**OR (95% CI)**	**OR (95% CI)**	**OR (95% CI)**	**OR (95% CI)**	**OR (95% CI)**
**Age**	1.04 [0.98, 1.10]	1.04 [0.98, 1.10]	1.03 [0.98, 1.09]	1.00 [0.94, 1.07]	1.02 [0.96, 1.08]	1.00 [0.94, 1.07]
**Men**	0.60 [0.41, 0.89]	0.39 [0.24, 0.63]	0.71 [0.48, 1.05]	0.45 [0.28, 0.75]	0.55 [0.36, 0.85]	0.43 [0.25, 0.72]
**Marital status (ref. Married/cohabiting)**						
Other	1.32 [0.99,1.75]	1.19 [0.87, 1.62]	1.29 [0.97, 1.71]	1.15 [0.84, 1.57]	1.18 [0.88, 1.60]	1.14 [0.82, 1.57]
**Education (ref. Higher education)**^**e**^						
Intermediate education	1.57 [1.11, 2.21]	2.27 [1.51, 3.40]	1.47 [1.03, 2.09]	2.16 [1.42, 3.27]	1.37 [0.95, 1.97]	2.05 [1.34, 3.15]
Basic education	2.10 [1.43, 3.09]	2.87 [1.83, 4.50]	1.91 [1.28, 2.86]	2.66 [1.67, 4.25]	1.59 [1.05, 2.43]	2.38 [1.47, 3.85]
**Physical workload (ref. Non-strenuous)**^**f**^						
Intermediate	0.94 [0.66, 1.33]	0.88 [0.60, 1.30]	0.92 [0.64, 1.30]	0.87 [0.59, 1.28]	1.00 [0.69, 1.45]	0.87 [0.58, 1.31]
Physically strenuous	1.41 [0.99, 2.01]	1.39 [0.95, 2.01]	1.19 [0.82, 1.72]	1.10 [0.74, 1.63]	1.32 [0.90, 1.95]	1.11 [0.74, 1.67]
**Sleep problems**^**g**^** (ref. No)**						
Occasional	2.11 [1.15, 3.84]	7.75 [2.45, 24.49]	2.08 [1.14, 3.81]	7.71 [2.43, 24.39]	1.96 [1.06, 3.62]	7.05 [2.21, 22.46]
Frequent	4.90 [2.50, 9.62]	14.76 [4.44, 49.06]	4.86 [2.47, 9.55]	14.68 [4.40, 48.90]	2.44 [1.16, 5.10]	6.64 [1.90, 23.27]
Missing	2.60 [1.27, 5.34]	7.17 [2.07, 24.83]	2.47 [1.20, 5.08]	6.75 [1.95, 23.43]	4.27 [2.13, 8.57]	12.61 [3.75, 42.47]
**Leisure-time physical activity**^**h**^** (LTPA) (ref. Vigorously active)**						
Moderately active	2.05 [1.20, 3.52]	4.74 [2.70, 8.35]	2.02 [1.18, 3.46]	2.40 [1.30, 4.41]	1.57 [0.91, 2.73]	1.89 [1.02, 3.51]
Inactive	3.97 [2.42, 6.50]	2.45 [1.33, 4.49]	3.99 [2.43, 6.54]	4.82 [2.73, 8.51]	2.23 [1.17, 4.28]	1.45 [0.64, 3.29]
Missing	2.56 [1.36, 4.82]	1.70 [0.76, 3.81]	2.56 [1.35, 4.83]	1.70 [0.76, 3.81]	2.50 [1.50, 4.17]	3.24 [1.81, 5.79]
**Alcohol consumption**^**i**^** (ref. No binge-drinking)**						
Binge-drinking	2.35 [1.57, 3.52]	1.62 [0.98, 2.67]	2.41 [1.61, 3.63]	1.68 [1.02, 2.78]	0.62 [0.40, 0.96]	0.79 [0.46, 1.34]
**Smoking**^**j**^** (ref. No)**						
Yes	1.98 [1.39, 2.80]	1.13 [0.72, 1.76]	1.84 [1.30, 2.63]	1.04 [0.66, 1.64]	1.84 [1.26, 2.70]	1.08 [0.69, 1.73]
**Fruit and vegetable consumption**^**k**^** (ref. Daily consumer)**						
Non-daily consumer	1.81 [1.37, 2.40]	1.30 [0.96, 1.76]	1.72 [1.30, 2.27]	1.22 [0.90, 1.66]	1.59 [1.19, 2.14]	1.15 [0.83, 1.58]
**Body mass index (BMI) (ref. Normal/healthy weight)**						
Overweight (BMI 25.0–29.9 kg/m2	1.50 [1.06, 2.11]	2.12 [1.46, 3.08]	1.44 [1.02, 2.89]	1.99 [1.37, 2.89]	1.38 [0.97, 1.95]	1.82 [1.24, 2.66]
Obesity (BMI ≥ 30 kg/m2)	5.44 [3.85, 7.68]	6.06 [4.10, 8.97]	5.21 [3.70, 7.37]	5.68 [3.83, 8.42]	4.65 [3.23, 6.70]	4.52 [3.00, 6.80]

^a^ Results are based on multinomial logistic regression analyses. Odds ratios (OR) with 95% confidence intervals (CI) are shown

^b^ Model 1 adjusted for age and gender

^c^ Model 1 + marital status, education, and physical workload

^d^ Model 2 + lifestyle-related factors and BMI

^e^ Higher education = university degree or more, intermediate education = matriculation or college education, basic education = primary or secondary school or less

^f^ Physically non-strenuous = very light, intermediate = rather light, physically strenuous = rather strenuous/very strenuous

^g^ Occasional = any symptoms in ≤ 14 nights/month, frequent = any symptoms in > 14 nights/month

^h^ Vigorously active = ≥ 14 metabolic equivalent task (MET) hours/week including the two highest intensity grades, moderately active = ≥ 14 MET hours/week including the two lowest intensity grades, inactive = < 14 MET hours/week

^i^ Binge-drinking = once a week or more, no binge-drinking = once a month or less

^j^ No = no current smoking, yes = current smoker

^k^ Daily consumer = consuming both fruit and vegetables daily, non-daily consumer = consuming fruit or vegetables less than daily

## Discussion

We sought to identify the developmental patterns of physical functioning before and after retirement among former Finnish municipal employees. Moreover, we examined how social- and health-related factors were associated with these physical functioning trajectories.

Distinct developmental patterns in physical functioning were found among ageing and retired public sector employees. Three distinct trajectory groups of physical functioning were selected: ‘fast decreasing’, ‘stable high’, and ‘slowly increasing’. When we regarded the time after retirement, we observed that the ‘slowly increasing’ trajectory reached the level of physical functioning with the ‘fast decreasing’ trajectory soon after retirement. Eventually, the ‘slowly increasing’ trajectory reached the level of physical functioning with the ‘stable high’ trajectory years after retirement. Lower educational level, sleep problems, and obesity were associated with the trajectory groups ‘fast decreasing’ and ‘slowly increasing’, in particular. Thus, the results suggest that these social- and health-related factors might increase the risk for declining physical functioning and lower levels of physical functioning in the baseline during the retirement period.

To the best of our knowledge, this was the first study examining physical functioning trajectories among statutory retirees, using a person-oriented approach. We were able to examine the developmental patterns of physical functioning both before and after statutory retirement. It should be noted that this study focused on statutory retirees, excluding participants in disability retirement, for example. Since the participants were able to work until statutory retirement, it indicates that the participants had, in general, decent physical functioning, and the healthy worker effect is probable. This might explain why the levels of physical functioning were relatively high in all three trajectory groups. Furthermore, the cohort was targeted to employed people and has little to say about non-employed people, who usually have poorer health [33–35].

Previous studies have suggested that lower education is associated with a risk of lower physical functioning [36–39]. This is in line with our findings since lower education was associated with both declining physical functioning and initially lower levels of physical functioning. Surprisingly, education as a socioeconomic factor did not explain the associations between health-related factors and the trajectories, even though it is known that education is associated with health-related factors [40, 41]. For example, better educated people are less likely to smoke, have high alcohol consumption, and be living with obesity compared to less educated people [40]. Then again, this study did not take into account the responders’ utilisation of preventive healthcare, resilience, psychological capital, and health literacy, which are suggested to be possible buffers against adverse socioeconomic circumstances [42, 43].

Poor health-related factors, including binge drinking, smoking, and non-daily F&V consumption, were more strongly associated with the ‘fast decreasing’ trajectory, which started with a high physical functioning level, than with the ‘slowly increasing’ trajectory, which started from a lower physical functioning level. While physical functioning generally declines with age, these findings support the fact that poor health-related factors might accelerate this process. Poor sleeping and obesity, which are both already known risk factors for poor physical functioning [16, 44], were strongly associated with the ‘fast decreasing’ and ‘slowly increasing’ trajectories. Stenholm, Leskinen & Viikari [45] suggested that, for example, removal of job strain, the possibility to increase LTPA and sleeping might result in improved functioning. They inform that previous studies have shown that the positive effects of retirement are greatest with individuals retiring from physically or mentally high job strain [46, 47]. This study did not clarify the possible corrective effect of the aforementioned associations, since for example mental workload was not considered in the analyses.

Surprisingly, working conditions were not associated with physical functioning trajectories. This might be due to the fact that this study only regarded those on statutory retirement; for example, impaired physical mobility is associated with earlier transition out of work [48]. The responders with the most adverse working conditions might have retired due to disability and thus were not considered in this study. Also, in this study, the physical working conditions were derived from the questionnaire prior to retirement. Hence, the analyses did not take into account how the physical workload through responders’ entire work career might affect physical functioning after retirement [49].

This study has a few limitations. First, the phase 1 responders were all municipal employees, hence the cohort does not represent the entire working population or general population. Due to that, the results might not be generalisable to other sectors or at the population level. Second, the covariates were measured prior to retirement. Hence, changes in social- and health-related factors during the follow-up were not considered. Third, most of the responders were women. It is likely that the trajectories and the covariates behind the trajectories would vary among women and men. However, the sample size among men was too small to analyse trajectories separately for women and men. Nevertheless, most of the employees in the Finnish municipal sector in general are women, which makes it possible to generalise the results to that population on statutory retirement. Also, like mentioned before, the healthy worker effect is possible, which can cause bias in the responses. Chronic diseases, among other health issues may force to retire earlier. Healthy workers tend to remain in the workforce. Also, workers usually remain employed because they are healthier but also have better access to healthcare [50, 51]. These factors might attenuate the results. The cohort profile of the Helsinki Health Study shows that men, those with younger age, those with poorer health, and those in lower occupational positions were less likely to respond to the baseline (2000–2002) and follow-up (2007) surveys [19]. Nevertheless, responses to our surveys in each phase were relatively good, and the previous non-response analyses indicated that the data represent satisfactorily the target population [19].

The main strength of this study is the longitudinal repeated data with four time points. This provided us the possibility to examine the trajectories of physical functioning years before and after retirement since the RAND-36 measure was repeated at each time point. Even though the physical functioning was self-reported, the RAND-36 measure is widely used and an established measure to study levels of physical functioning [52, 22]. The longitudinal data was rich including a high number of participants and included multiple covariates that are known to be risk factors for poor physical functioning. Trajectory analysis is found to be a strong tool to examine heterogeneity, discover new patterns, and connect these to covariates. However, it should be kept in mind that the identified trajectories are approximations of the actual developmental patterns [53].

We suggest that further studies should consider measuring social- and health-related factors after retirement to examine whether changes in social- and health-related factors after retirement could explain the patterns of the physical functioning trajectories years after retirement.

## Conclusion

This study shows that lower education and health-related risk factors were associated with trajectories of lower levels of physical functioning before retirement and declining physical functioning after statutory retirement. In particular, physical inactivity, sleep problems, and obesity were associated with declining and lower-level physical functioning trajectories. Future studies could investigate whether changes in these or other social- and health-related factors during the retirement period impact the trajectories of physical functioning after retirement since we observed both increasing and decreasing patterns in physical functioning years after retirement.

This would provide important knowledge to plan targeted interventions for both the employees prior to their retirement, and to assess whether similar interventions are needed after retirement. Interventions which aim to support and improve healthy lifestyles among older employees, especially among those with lower education, are recommended to help the population to maintain better physical functioning and possibly lengthen their work careers.

## Supplementary Information

Supplementary Material 1.

## Data Availability

The datasets generated and/or analysed during the current study are not publicly available due to data protection laws but are available from the corresponding author on reasonable request.

## Abbreviations

AIC: Akaike information criterion
BIC: Bayesian information criterion
BMI: Body mass index
CI: Confidence interval
GMM: Growth mixture modelling
F&V: Fruit and vegetable
FFQ: Food frequency questionnaire
LTPA: Leisure-time physical activity
MET: Metabolic equivalent task
OR: Odds ratio

## References

[1] Eurostat: Increase in the share of the population aged 65 years or over between 2011 and 2021. https://ec.europa.eu/eurostat/statistics-explained/index.php?title=Population_structure_and_ageing. Accessed 26 Jan 2023.

[2] Eurofound: Ageing workforce. https://www.eurofound.europa.eu/topic/ageing-workforce. Accessed 26 Jan 2023.

[3] Saha P, Salmela J, Lallukka T, Aho AL. Functioning Changes in Varying Ways After Retirement: A Scoping Review. Inquiry. 2023. 10.1177/00469580221142477. 36604784 10.1177/00469580221142477 PMC9830080

[4] Lahelma E, Pietiläinen O, Chandola T, Hyde M, Rahkonen O, Lallukka T. Occupational social class trajectories in physical functioning among employed women from midlife to retirement. BMC Public Health. 2019;19(1):1525. 31727156 10.1186/s12889-019-7880-0 PMC6857143

[5] Stenholm S, Westerlund H, Salo P, Hyde M, Pentti J, Head J, et al. Age-related trajectories of physical functioning in work and retirement: the role of sociodemographic factors, lifestyle and disease. J Epidemiol Community Health. 2014;68(6):503–9. 24534071 10.1136/jech-2013-203555

[6] van Zon SKR, Bültmann U, Reijneveld SA, de Leon CFM. Functional health decline before and after retirement: A longitudinal analysis of the Health and Retirement Study. Soc Sci Med. 2016;170:26–34. 27741444 10.1016/j.socscimed.2016.10.002

[7] Nilsen C, Nelson ME, Andel R, Crowe M, Finkel D, Pedersen NL. Job Strain and Trajectories of Cognitive Change Before and After Retirement. J Gerontol B Psychol Sci Soc Sci. 2021;76(7):1313–22. 33624114 10.1093/geronb/gbab033 PMC8363035

[8] Sabbath EL, Andel R, Zins M, Goldberg M, Berr C. Domains of cognitive function in early old age: which ones are predicted by pre-retirement psychosocial work characteristics? Occup Environ Med. 2016;73(10):640–7. 27188277 10.1136/oemed-2015-103352 PMC5429340

[9] Biernat E, Skrok Ł, Krzepota J. Short-Term and Medium-Term Impact of Retirement on Sport Activity, Self-Reported Health, and Social Activity of Women and Men in Poland. Biomed Res Int. 2019. 10.1155/2019/8383540. 31111069 10.1155/2019/8383540 PMC6487168

[10] Roberts BA, Fuhrer R, Marmot M, Richards M. Does retirement influence cognitive performance? The Whitehall II Study. J Epidemiol Community Health. 2011;65(11):958–63. 20940172 10.1136/jech.2010.111849

[11] Ihle A, Grotz C, Adam S, Oris M, Fagot D, Gabriel R, et al. The association of timing of retirement with cognitive performance in old age: the role of leisure activities after retirement. Int Psychogeriatr. 2016;28(10):1659–69. 27378103 10.1017/S1041610216000958

[12] Bauger L, Bongaardt R. The lived experience of well-being in retirement: A phenomenological study. Int J Qual Stud Health Well-being. 2016. 10.3402/qhw.v11.33110. 27814778 10.3402/qhw.v11.33110 PMC5097159

[13] Platts LG, Webb E, Zins M, Goldberg M, Netuveli G. Mid-life occupational grade and quality of life following retirement: a 16-year follow-up of the French GAZEL study. Aging Ment Health. 2015;19(7):634–46. 25220504 10.1080/13607863.2014.955458 PMC4396408

[14] Ko SU, Hausdorff JM, Ferrucci L. Age-associated differences in the gait pattern changes of older adults during fast-speed and fatigue conditions: Results from the Baltimore longitudinal study of ageing. Age Ageing. 2010;39(6):688–94. 20833863 10.1093/ageing/afq113 PMC2956533

[15] Ferrucci L, Cooper R, Shardell M, Simonsick EM, Schrack JA, Kuh D. Age-related change in mobility: Perspectives from life course epidemiology and geroscience. J Gerontol A Biol Sci Med Sci. 2016;71(9):1184–94. 26975983 10.1093/gerona/glw043 PMC4978365

[16] Lallukka T, Hiilamo A, Pietiläinen O, Mänty M, Kouvonen A, Rahkonen O. Who maintains good health functioning? The contribution of social, work-related and behavioural factors to mental and physical health functioning trajectories in ageing employees. Occup Environ Med. 2020;77(7):478–87. 32201385 10.1136/oemed-2019-106324

[17] Sabbath EL, Glymour MM, Descatha A, Leclerc A, Zins M, Goldberg M, et al. Biomechanical and psychosocial occupational exposures: joint predictors of post-retirement functional health in the French GAZEL cohort. Adv Life Course Res. 2013;18(4):235–43. 24796708 10.1016/j.alcr.2013.07.002

[18] Laitalainen E, Helakorpi S, Martelin T, Uutela A. Eläkeikäisten toimintakyky on parantunut, mutta ei kaikissa väestöryhmissä (in Finnish). Lääkärilehti. 2010;65(41):3295–301.

[19] Lahelma E, Aittomäki A, Laaksonen M, Lallukka T, Martikainen P, Piha K, et al. Cohort Profile: The Helsinki Health Study. Int J Epidemiol. 2013;42(3):722–30. 22467288 10.1093/ije/dys039

[20] Hays RD, Sherbourne CD, Mazel RM. The rand 36-item health survey 1.0. Health Econ. 1993;2(3):217–27. 10.1002/hec.4730020305 8275167

[21] Aalto AM, Aro AR, Teperi J. RAND-36 terveyteen liittyvän elämänlaadun mittarina : mittarin luotettavuus ja suomalaiset väestöarvot (in Finnish). Helsinki: Stakes; 1999. https://urn.fi/URN:NBN:fi-fe201211089642. Accessed 11 Sep 2023.

[22] Hays RD, Morales LS. The RAND-36 measure of health-related quality of life. Ann Med. 2001;33(5):350–7. 11491194 10.3109/07853890109002089

[23] Lahti J, Lallukka T, Harkko J, Nordquist H, Mänty M, Pietiläinen O, et al. Working conditions and antidepressant medication use: A prospective study among 18 to 39-year-old municipal employees. Psychiatry Res. 2021. 10.1016/j.psychres.2021.114213. 34563974 10.1016/j.psychres.2021.114213

[24] Salonsalmi A, Rahkonen O, Lahelma E, Laaksonen M. The association between alcohol drinking and self-reported mental and physical functioning: a prospective cohort study among City of Helsinki employees. BMJ Open. 2017;7(4): e014368. 28473511 10.1136/bmjopen-2016-014368 PMC5623368

[25] Jenkins CD, Stanton BA, Niemcryk SJ, Rose RM. A scale for the estimation of sleep problems in clinical research. J Clin Epidemiol. 1988;41(4):313–21. 3351539 10.1016/0895-4356(88)90138-2

[26] Shahid A, Wilkinson K, Marcu S, Shapiro CM. Jenkins Sleep Scale. In: STOP, THAT and One Hundred Other Sleep Scales. New York, NY: Springer New York; 2011. p. 203–4.

[27] Holstila A, Mänty M, Rahkonen O, Lahelma E, Lahti J. Changes in leisure-time physical activity and physical and mental health functioning: a follow-up study. Scand J Med Sci Sports. 2017;27(12):1785–92. 27714910 10.1111/sms.12758

[28] Lahti J, Holstila A, Lahelma E, Rahkonen O. Leisure-time physical activity and all-cause mortality. PLoS ONE. 2014;9(7): e101548. 24988295 10.1371/journal.pone.0101548 PMC4079687

[29] Ram N, Grimm KJ. Methods and Measures: Growth mixture modeling: A method for identifying differences in longitudinal change among unobserved groups. Int J Behav Dev. 2009;33(6):565–76. 23885133 10.1177/0165025409343765 PMC3718544

[30] Herle M, Micali N, Abdulkadir M, Loos R, Bryant-Waugh R, Hübel C, et al. Identifying typical trajectories in longitudinal data: modelling strategies and interpretations. Eur J Epidemiol. 2020;35(3):205–22. 32140937 10.1007/s10654-020-00615-6 PMC7154024

[31] Proust-Lima C, Philipps V, Liquet B. Estimation of Extended Mixed Models Using Latent Classes and Latent Processes: The R Package lcmm. J Stat Softw. 2017;78(2):1–56.

[32] Nagin DS, Odgers CL. Group-based trajectory modeling in clinical research. Annu Rev Clin Psychol. 2010;6:109–38. 20192788 10.1146/annurev.clinpsy.121208.131413

[33] Bartley M, Sacker A, Clarke P. Employment status, employment conditions, and limiting illness: prospective evidence from the British household panel survey 1991–2001. J Epidemiol Community Health. 2004;58(6):501–6. 15143119 10.1136/jech.2003.009878 PMC1732781

[34] Martikainen P, Valkonen T. Bias related to the exclusion of the economically inactive in studies on social class differences in mortality. Int J Epidemiol. 1999;28(5):899–904. 10597989 10.1093/ije/28.5.899

[35] McKee-Ryan FM, Song Z, Wanberg CR, Kinicki AJ. Psychological and Physical Well-Being During Unemployment: A Meta-Analytic Study. J Appl Psychol. 2005;90(1):53–76. 15641890 10.1037/0021-9010.90.1.53

[36] Audureau E, Rican S, Coste J. Worsening trends and increasing disparities in health-related quality of life: evidence from two French population-based cross-sectional surveys, 1995–2003. Qual Life Res. 2013;22(1):13–26. 22298202 10.1007/s11136-012-0117-7

[37] Pietiläinen O, Laaksonen M, Pitkäniemi J, Rahkonen O, Lahelma E. Changes of occupational class differences in physical functioning: a panel study among employees (2000–2007). J Epidemiol Community Health. 2012;66(3):265–70. 20924055 10.1136/jech.2010.110270

[38] Stefler D, Prina M, Wu YT, Sánchez-Niubò A, Lu W, Haro JM, et al. Socioeconomic inequalities in physical and cognitive functioning: cross-sectional evidence from 37 cohorts across 28 countries in the ATHLOS project. J Epidemiol Community Health. 2021;75(10):980–6. 33649052 10.1136/jech-2020-214714

[39] Van Dyck D, Cardon G, De Bourdeaudhuij I. Longitudinal changes in physical activity and sedentary time in adults around retirement age: what is the moderating role of retirement status, gender and educational level? BMC Public Health. 2016;16(1):1125. 27793134 10.1186/s12889-016-3792-4 PMC5084354

[40] Brunello G, Fort M, Schneeweis N, Winter-Ebmer R. The Causal Effect of Education on Health: What is the Role of Health Behaviors? Health Econ. 2016;25(3):314–36. 25581162 10.1002/hec.3141

[41] Cutler DM, Lleras-Muney A. Understanding differences in health behaviors by education. J Health Econ. 2010;29(1):1–28. 19963292 10.1016/j.jhealeco.2009.10.003 PMC2824018

[42] Massar K, Kopplin N, Schelleman-Offermans K. Childhood Socioeconomic Position, Adult Educational Attainment and Health Behaviors: The Role of Psychological Capital and Health Literacy. Int J Environ Res Public Health. 2021;18(17):9399. 34501988 10.3390/ijerph18179399 PMC8430706

[43] Perna L, Mielck A, Lacruz ME, Emeny RT, Holle R, Breitfelder A, et al. Socioeconomic position, resilience, and health behaviour among elderly people. Int J Public Health. 2012;57(2):341–9. 21912944 10.1007/s00038-011-0294-0

[44] Svärd A, Lahti J, Roos E, Rahkonen O, Lahelma E, Lallukka T, et al. Obesity, change of body mass index and subsequent physical and mental health functioning: a 12-year follow-up study among ageing employees. BMC Public Health. 2017;17(1):744. 28950839 10.1186/s12889-017-4768-8 PMC5615472

[45] Stenholm S, Leskinen T, Viikari L. Eläköityneiden terveyden edistämiseen kannattaa panostaa (in Finnish). Duodecim. 2019;135(11):1068–74.

[46] Schuring M, Robroek SJ, Lingsma HF, Burdorf A. Educational differences in trajectories of self-rated health before, during, and after entering or leaving paid employment in the European workforce. Scand J Work Environ Health. 2015;41(5):441–50. 26186611 10.5271/sjweh.3514

[47] Westerlund H, Kivimäki M, Singh-Manoux A, Melchior M, Ferrie JE, Pentti J, et al. Self-rated health before and after retirement in France (GAZEL): a cohort study. Lancet. 2009;374(9705):1889–96. 19897238 10.1016/S0140-6736(09)61570-1

[48] Rice NE, Lang IA, Henley W, Melzer D. Common health predictors of early retirement: findings from the English Longitudinal Study of Ageing. Age Ageing. 2011;40(1):54–61. 21148324 10.1093/ageing/afq153

[49] Sundstrup E, Hansen ÅM, Mortensen EL, Poulsen OM, Clausen T, Rugulies R, et al. Retrospectively assessed physical work environment during working life and risk of sickness absence and labour market exit among older workers. Occup Environ Med. 2018;75(2):114–23. 28819019 10.1136/oemed-2016-104279 PMC5800344

[50] Chowdhury R, Shah D, Payal AR. Healthy Worker Effect Phenomenon: Revisited with Emphasis on Statistical Methods - A Review. Indian journal of occupational and environmental medicine. 2017;21(1):2–8. 29391741 10.4103/ijoem.IJOEM_53_16 PMC5763838

[51] Neophytou AM, Picciotto S, Hart JE, Garshick E, Eisen EA, Laden F. A structural approach to address the healthy-worker survivor effect in occupational cohorts: an application in the trucking industry cohort. Occupational and environmental medicine (London, England). 2014;71(6):442–7. 10.1136/oemed-2013-102017 PMC4051133 24727736

[52] Bohannon RW, DePasquale L. Physical Functioning Scale of the Short-Form (SF) 36: internal consistency and validity with older adults. J Geriatr Phys Ther. 2010;33(1):16–8. 20503729

[53] Serra L, Farrants K, Alexanderson K, Ubalde M, Lallukka T. Trajectory analyses in insurance medicine studies: Examples and key methodological aspects and pitfalls. PLoS ONE. 2022;17(2): e0263810. 35148351 10.1371/journal.pone.0263810 PMC8836370

