# Supplementary material for: Physical functioning trajectories over statutory retirement: a finnish occupational cohort study
Source: Arch Public Health. 2025 Jan 10;83:8. doi: 10.1186/s13690-024-01483-2 (PMC11720325; doi:10.1186/s13690-024-01483-2)
Supplement: Supplementary file 1 — Supplementary Material 1. [file 13690_2024_1483_MOESM1_ESM.docx]

SUPPLEMENTAL MATERIAL

**Supplementary Table 1: Model fit statistics for trajectory modelling**

|  |  | Model |  |  |  |
| --- | --- | --- | --- | --- | --- |
|  |  | m1 | m2 | m3 | m4 |
| Number of latent classes | | 1.00 | 2.00 | 3.00 | 4.00 |
| Maximum log-likelihood | | -43896.44 | -43544.25 | -43329.43 | -43178.48 |
| AIC |  | 87812.89 | 87116.49 | 86694.86 | 86400.95 |
| BIC |  | 87872.03 | 87199.29 | 86801.32 | 86531.06 |
| Entropy |  | 1.00 | 0.90 | 0.88 | 0.90 |
| % Group 1 | (N) | 100.00 | 9.758772 (N=267) | 8.479532 (N=232) | 6.871345 (N=188) |
| % Group 2 | (N) |  | 90.24123 (N=2469) | 84.32 (N=2307) | 6.980994 (N=191) |
| % Group 3 | (N) |  |  | 7.2 (N=197) | 83.479532 (N=2284) |
| % Group 4 | (N) |  |  |  | 2.668129 (N=73) |
| Mean of posterior probabilities | | |  |  |  |
| probability 1 | | - | 0.90 | 0.85 | 0.97 |
| probability 2 | | - | 0.98 | 0.97 | 0.80 |
| probability 3 | | - | - | 0.88 | 0.86 |
| probability 4 | | - | - | - | 0.87 |
| Posterior probabilities above threshold >0.7 (%) | | | | |  |
| Class 1 |  | - | 85.77 | 75.86 | 96.63 |
| Class 2 |  | - | 98.26 | 97.01 | 64.38 |
| Class 3 |  | - | - | 81.22 | 78.01 |
| Class 4 |  | - | - | - | 81.38 |

**Supplementary Table 2: Associations of covariates with physical functioning trajectories, odds ratios with 95% confidence intervals from multinomial logistic regression models.**

|  | **Model 1**^a^ |  | **Model 2**^b^ |  | **Model 3^c^** |  | **Model 4^d^** |  | **Model 5^e^** |  |
| --- | --- | --- | --- | --- | --- | --- | --- | --- | --- | --- |
|  | **Group 1 - fast decreasing** | **Group 3 - slowly increasing** | **Group 1 - fast decreasing** | **Group 3 - slowly increasing** | **Group 1 - fast decreasing** | **Group 3 - slowly increasing** | **Group 1 - fast decreasing** | **Group 3 - slowly increasing** | **Group 1 - fast decreasing** | **Group 3 - slowly increasing** |
|  | **OR (95% CI)** | **OR (95% CI)** | **OR (95% CI)** | **OR (95% CI)** | **OR (95% CI)** | **OR (95% CI)** | **OR (95% CI)** | **OR (95% CI)** | **OR (95% CI)** | **OR (95% CI)** |
| **Age** | 1.04 [0.98, 1.10] | 1.04 [0.98, 1.10] | 1.04 [0.98, 1.10] | 1.03 [0.98, 1.09] | 1.03 [0.98, 1.09] | 1.00 [0.94, 1.07] | 1.03 [0.98, 1.09] | 1.00 [0.94, 1.08] | 1.02 [0.96, 1.08] | 1.00 [0.94, 1.07] |
| **Men** | 0.60 [0.41, 0.89] | 0.39 [0.24, 0.63] | 0.64 [0.44, 0.94] | 0.40 [0.25, 0.66] | 0.71 [0.48, 1.05] | 0.45 [0.28, 0.75] | 0.71 [0.48, 1.05] | 0.45 [0.28, 0.75] | 0.55 [0.36, 0.85] | 0.43 [0.25, 0.72] |
| **Marital status (ref. Married/cohabiting)**  Other | 1.32 [0.99 ,1.75] | 1.19 [0.87, 1.62] | 1.32 [0.99, 1.75] | 1.19 [0.87, 1.61] | 1.29 [0.97, 1.71] | 1.15 [0.84, 1.57] | 1.29 [0.97, 1.71] | 1.17 [0.97, 1.09] | 1.18 [0.88, 1.60] | 1.14 [0.82, 1.57] |
| **Education^f^ (ref. Higher education)**  Intermediate education  Basic education | 1.57 [1.11, 2.21]  2.10 [1.43, 3.09] | 2.27 [1.51, 3.40]  2.87 [1.83, 4.50] | 1.55 [1.10, 2.19]  2.07 [1.41, 3.05] | 2.26 [1.50, 3.39]  2.85 [1.81, 4.47] | 1.47 [1.03, 2.09]  1.91 [1.28, 2.86] | 2.16 [1.42, 3.27]  2.66 [1.67, 4.25] | 1.47 [1.03, 2.09]  1.91 [1.28, 2.86] | 2.16 [1.42, 3.27]  2.66 [1.67, 4.25] | 1.37 [0.95, 1.97]  1.59 [1.05, 2.43] | 2.05 [1.34, 3.15]  2.38 [1.47, 3.85] |
| **Physical workload^g^ (ref. Non-strenuous)**  Intermediate  Physically strenuous | 0.94 [0.66, 1.33]  1.41 [0.99, 2.01] | 0.88 [0.60, 1.30]  1.39 [0.95, 2.01] | 0.93 [0.65, 1.32]  1.39 [0.97, 1.98] | 0.88 [0.60, 1.29]  1.38 [0.94, 2.02] | 0.92 [0.64, 1.30]  1.19 [0.82, 1.72] | 0.87 [0.59, 1.28]  1.10 [0.74, 1.63] | 0.92 [0.64, 1.30]  1.19 [0.82, 1.72] | 0.87 [0.59, 1.28]  1.10 [0.74, 1.63] | 1.00 [0.69, 1.45]  1.32 [0.90, 1.95] | 0.87 [0.58, 1.31]  1.11 [0.74, 1.67] |
| **Sleep problems^h^ (ref. no)**  Occasional  Frequent  Missing | 2.11 [1.15, 3.84]  4.90 [2.50, 9.62]  2.60 [1.27, 5.34] | 7.75 [2.45, 24.49]  14.76 [4.44, 49.06]  7.17 [2.07, 24.83] | 2.11 [1.15, 3.85]  4.90 [2.50, 9.61]  2.63 [1.28, 5.39] | 7.76 [2.45, 24.52]  14.77 [4.44, 49.10]  7.22 [2.08, 25.01] | 2.11 [1.16, 3.86]  4.94 [2.52, 9.71]  2.52 [1.23, 5.18] | 7.78 [2.46, 24.63]  14.95 [4.49, 49.78]  6.84 [1.97, 23.74] | 2.08 [1.14, 3.81]  4.86 [2.47, 9.55]  2.47 [1.20, 5.08] | 7.71 [2.43, 24.39]  14.68 [4.40, 48.90]  6.75 [1.95, 23.43] | 1.96 [1.06, 3.62]  2.44 [1.16, 5.10]  4.27 [2.13, 8.57] | 7.05 [2.21, 22.46]  6.64 [1.90, 23.27]  12.61 [3.75, 42.47] |
| **Leisure-time physical activity (LTPA)^i^ (ref. Vigorously active)**  Moderately active  Inactive  Missing | 2.05 [1.20, 3.52]  3.97 [2.42, 6.50]  2.56 [1.36, 4.82] | 4,74 [2,70, 8,35]  2,45 [1,33, 4,49]  1,70 [0,76, 3,81] | 2.07 [1,21, 3,54]  3.99 [2.44, 6.54]  2.59 [1.37, 4.88] | 2.46 [1.34, 4.51]  4.76 [2.71, 8.38]  1.71 [0.76, 3.84] | 2.04 [1.19, 3.49]  4.01 [2.45, 6.58]  2.56 [1.36, 4.84] | 2,02 [1,18, 3,46]  3,99 [2,43, 6,54]  2,56 [1,35, 4,83] | 2.02 [1.18, 3.46]  3.99 [2.43, 6.54]  2.56 [1.35, 4.83] | 2.40 [1.30, 4.41]  4.82 [2.73, 8.51]  1.70 [0.76, 3.81] | 1.57 [0.91, 2.73]  2.23 [1.17, 4.28]  2.50 [1.50, 4.17] | 1.89 [1.02, 3.51]  1.45 [0.64, 3.29]  3.24 [1.81, 5.79] |
| **Alcohol consumption^j^ (ref. No binge-drinking)**  Binge-drinking | 2.35 [1.57, 3.52] | 1.62 [0.98, 2.67] | 2.34 1.56, 3.51] | 1.61 [0.98, 2.66] | 2.41 [1.61, 3.63] | 1.68 [1.02, 2.78] | 2.47 [1.64, 3.71] | 1.70 [1.03, 2.82] | 0.62 [0.40, 0.96] | 0.79 [0.46, 1.34] |
| **Smoking^k^ (ref. no)**  Yes | 1.98 1.39, 2.80] | 1.13 [0.72, 1.76] | 1.93 [1.36, 2.74] | 1.11 [0.71, 1.74] | 1.84 [1.30, 2.63] | 1.04 [0.66, 1.64] | 1.85 [1.30, 2.64] | 1.04 [0.66, 1.64] | 1.84 [1.26, 2.70] | 1.08 [0.67, 1.74] |
| **Fruit and Vegetable Consumption^l^ (ref. Daily consumer)**  Non-daily | 1.81 [1.37, 2.40] | 1.30 [0.96, 1.76] | 1.78 [1.35, 2.35] | 1.29 [0.95, 1.74] | 1.72 [1.30, 2.27] | 1.22 [0.90, 1.66] | 1.72 [1.30, 2.27] | 1.22 [0.90, 1.66] | 1.59 [1.19, 2.14] | 1.15 [0.83, 1.58] |
| **BMI (ref. Normal/healthy weight)**  Overweight (BMI 25.0–29.9 kg/m^2^)  Obesity (BMI ≥ 30 kg/m^2^) | 1.50 [1.06, 2.11]  5.44 [3.85, 7.68] | 2.12 [1.46, 3.08]  6.06 [4.10, 8.97] | 1.51 [1.07, 2.12]  5.41 [3.83, 7.64] | 2.13 [1.47, 3.09]  6.04 [4.08, 8.94] | 1.44 [1.02, 2.89]  5.21 [3.70, 7.37] | 1.99 [1.37, 2.89]  5.68 [3.83, 8.42] | 1.44 [1.02, 2.03]  5.25 [3.71, 7.44] | 1.99 [1.37, 2.89]  5.73 [3.87, 8.50] | 1.38 [0.97, 1.95]  4.65 [3.23, 6.70] | 1.82 [1.24, 2.66]  4.52 [3.00, 6.80] |

^a^ age, gender

^b^ Model 1 + marital status

^c^ Model 2 + education

^d^ Model 3 + physical workload

^e^ Model 4 + lifestyle-related factors, BMI

^f^ Higher education = university degree or more, intermediate education = matriculation or college education, basic education = primary or secondary school or less

^g^ Physically non-strenuous = very light, Intermediate = rather light, Physically strenuous = rather strenuous/very strenuous

^h^ Occasional = any symptoms in ≤14 nights/month; frequent = any symptoms in >14 nights/month

^i^ Vigorously active = ≥14 metabolic equivalent task (MET) hours/week including the two highest intensity grades, moderately active = ≥14 MET hours/week including the two lowest intensity grades, inactive = <14 MET hours/week

^j^ Binge-drinking = once a week or more, no binge-drinking = once a month or less

^k^ No = no current smoker, Yes = current smoker

^l^ Daily consumer = consuming both fruit and vegetables daily, non-daily consumer = consuming fruit or vegetables less than daily

**Supplementary Table 3: Background characteristics of the study population**

|  | Women (%) | Men (%) | Total (%) |
| --- | --- | --- | --- |
| **Age (mean, SD)**  Mean  Standard deviation | 60.14  2.530 | 60.01  2.774 | 60.11  2.580 |
| **Marital status** |  |  |  |
| Married/cohabiting | 1414 (64.7) | 468 (85.1) | 1882 (68.8) |
| Other | 772 (35.3) | 82 (14.9) | 854 (31.2) |
| **Education^a^** |  |  |  |
| Higher education  Basic education | 631 (28.9)  473 (21.6) | 228 (41.5)  101 (18.4) | 859 (31.4)  574 (21.0) |
| Intermediate education | 1082 (49.5) | 221 (40.2) | 1303 (47.6) |
|  |  |  |  |
| **Physical workload^b^** |  |  |  |
| Physically non-strenuous | 529 (24.2) | 248 (45.1) | 777 (28.4) |
| Intermediate | 879 (40.2) | 236 (42.9) | 1115 (40.8) |
| Physically strenuous | 778 (35.6) | 66 (12.0) | 844 (30.8) |
| **Sleep problems^c^** |  |  |  |
| No  Occasional  Frequent  Missing | 210 (9.6)  1562 (71.5)  212 (9.7)  202 (9.2) | 78 (14.2)  394 (71.6)  38 (6.9)  40 (7.3) | 288 (10.5)  1956 (71.5)  250 (9.1)  242 (8.8) |
| **Leisure-time physical activity (LTPA)^d^**  Vigorously active  Moderately active  Inactive  Missing | 393 (18.0)  642 (29.4)  946 (43.3)  205 (9.4) | 155 (28.2)  121 (22.0)  216 (39.3)  58 (10.5) | 548 (20.0)  763 (27.9)  1162 (42.5)  263 (9.6) |
| **Alcohol consumption^e^** |  |  |  |
| No binge-drinking | 2055 (94.0) | 432 (78.5) | 2487 (90.9) |
| Binge-drinking | 131 (6.0) | 118 (21.5) | 249 (9.1) |
| **Smoking^f^** |  |  |  |
| No | 1935 (88.5) | 550 (84.7) | 2401 (87.8) |
| Yes | 251 (11.5) | 84 (15.3) | 335 (12.2) |
| **Fruit and vegetable consumption^g^** |  |  |  |
| Daily | 1471 (67.3) | 227 (41.3) | 1698 (62.1) |
| Non-daily | 715 (32.7) | 323 (58.7) | 1038 (37.9) |
| **BMI** |  |  |  |
| Normal weight (BMI <25 kg/m^2^) | 1021 (46.7) | 216 (39.3) | 1237 (45.2) |
| Overweight (BMI 25.0–29.9 kg/m^2^) | 810 (37.1) | 245 (44.5) | 1055 (38.6) |
| Obesity (BMI ≥ 30 kg/m^2^) | 355 (16.2) | 89 (16.2) | 444 (16.2) |
| **Physical functioning** | **Women** | **Men** | **Total** |
| Phase 1 | 87.629422 (N=2183) | 91.1404 (N=549) | 88.335212 (N=2732) |
| Phase 2 | 84.500102 (N=2186) | 89.8280 (N=549) | 85.571140 (N=2735) |
| Phase 3 | 84.500102 (N=2185) | 88.3291 (N=549) | 84.034007 (N=2734) |
| Phase 4 | 79.589914 (N=2180) | 84.7430 (N=547) | 80.625812 (N=2727) |

^a^ Higher education = university degree or more, intermediate education = matriculation or college education, basic education = primary or secondary school or less

^b^ Physically non-strenuous = very light, Intermediate = rather light, Physically strenuous = rather strenuous/very strenuous

^c^ Occasional = any symptoms in ≤14 nights/month; frequent = any symptoms in >14 nights/month

^d^ Vigorously active = ≥14 metabolic equivalent task (MET) hours/week including the two highest intensity grades, moderately active = ≥14 MET hours/week including the two lowest intensity grades, inactive = <14 MET hours/week

^e^ Binge-drinking = once a week or more, no binge-drinking = once a month or less

^f^ No = no current smoker, Yes = current smoker

^g^ Daily consumer = consuming both fruit and vegetables daily, non-daily consumer = consuming fruit or vegetables less than daily

**Supplementary Figure 1: Participant selection criteria**

**Analytical Sample**

**N=2736**

Excluded

N=1473:

Missing information on physical functioning in two or more phases (n=1017), missing information on the timing or type of retirement (n=449) or inconsistent information on the timing of retirement (n=7).

Target population

(40–60-year-old employees of the City of Helsinki, Finland; 2000–2002)

N=13,344

Other than statutory retired

N=4751

Statutory retired during the follow-up (2000-2017)

N=4209

Responders

N=8960 (67 %)
